# Development of SARS vaccines and therapeutics is still needed

**DOI:** 10.2217/fvl.12.126

**Published:** 2012-12-20

**Authors:** Shibo Jiang, Lu Lu, Lanying Du

**Affiliations:** 1 Key Laboratory of Medical Molecular Virology of Ministries of Education & Health, Shanghai Medical College & Institute of Medical Microbiology, Fudan University, Shanghai 200032, China; 2 The Lindsley F Kimball Research Institute, The New York Blood Center, New York, NY 10065, USA; 1 Key Laboratory of Medical Molecular Virology of Ministries of Education & Health, Shanghai Medical College & Institute of Medical Microbiology, Fudan University, Shanghai 200032, China. shibojiang@fudan.edu.cn

**Keywords:** HCoV-EMC, human coronavirus, receptor-binding domain, SARS, SARS-CoV, spike protein, therapeutics, vaccines

On 22 September 2012, the WHO was informed by the UK of a case of acute respiratory syndrome with renal failure [101]. The patient had a travel history to the Kingdom of Saudi Arabia, where a 60 year-old Saudi national died from a similar disease earlier in 2012. The causative pathogen, a novel human *Betacoronavirus* (HCoV-EMC), was quickly identified from the sputum samples of the earlier case [1] and the gene sequence of this virus was 99.5% identical to that of the virus isolated from clinical samples of the later case.

The outbreak of HCoV-EMC infection in Saudi Arabia has raised great concerns about the potential pandemic of the SARS-like disease, and strategies for combating this newly emerged infectious disease should be prepared [2]. It is believed that the existing SARS research may provide a useful template for developing vaccines and therapeutics against HCoV-EMC infection [3], but so far, no effective anti-SARS vaccines and therapeutics have been well developed.

SARS, which is caused by the SARS coronavirus (SARS-CoV), emerged from China and caused nearly 8500 cases and 916 deaths during the outbreak in 2002 and 2003 [3–5]. Although SARS is currently under control, the possibility of a new SARS outbreak remains a global concern because of the potential for zoonotic transmission of SARS-CoV or SARS-CoV-like viruses from their natural hosts to humans, or the accidental or intentional release of laboratory SARS-CoV strains [6]. Therefore, developing vaccines and therapeutics for the prevention and treatment of SARS is still a matter of urgency.

During the global 2002/2003 SARS pandemic, many foundations, pharmaceutical companies and governments provided abundant funds to support the development of anti-SARS vaccines and therapeutics. However, after the disappearance of SARS, these funds were either withdrawn or discontinued because of the lack of a sustainable market of the products to be developed.

A number of inactivated and live-attenuated SARS vaccines, as well as those based on vectors encoding the full-length S protein of SARS-CoV, showed high immunogenicity in inducing neutralizing antibody responses and protection against SARS-CoV challenge [6]. However, most of these vaccine candidates may also induce immunopathology or other harmful immune responses [7,8], raising concerns about their safety. On the other hand, recombinant proteins containing the receptor-binding domain of the SARS-CoV S protein could be developed as a safe and effective SARS vaccine. This potential is based on the ability of receptor binding domain-based vaccine to induce stronger cross-neutralizing antibody responses and protection against SARS-CoV [9], with a correspondingly lower probability of inducing immunopathology, in contrast with the other SARS vaccine candidates mentioned previously [6].

So far, no specific anti-SARS drugs have been developed. A number of drug candidates, including the SARS-CoV fusion inhibitors [10], proteinase (e.g., 3C-like cysteine proteinase) inhibitors, PLpro inhibitors, RNA-dependent RNA polymerase inhibitors, helicase inhibitors, siRNAs inhibiting SARS-CoV structural proteins E, M and N, and therapeutic antibodies, have been developed in laboratory and preclinical studies [11]. However, none of them have been forwarded to clinical trials, possibly for the same reasons as previously described for vaccine development.

Apart from financial support, another main challenge for the clinical development of anti-SARS vaccines is the lack of endemic SARS and the lethal nature of the disease. It is not ethical to conduct human efficacy studies by exposing healthy human volunteers to a lethal agent like SARS-CoV. Fortunately, however, according to the ‘Animal Rule’, a pivotal animal efficacy study can be conducted for evaluating the *in vivo* efficacy of the anti-SARS vaccines using two animal species that exhibit pathophysiology of the disease and host immune responses that closely match those of humans [12]. The data from these animal experiments can then be considered by the US FDA as evidence of effectiveness of the tested vaccine.
